# The Role of Maternal HIV Envelope-Specific Antibodies and Mother-to-Child Transmission Risk

**DOI:** 10.3389/fimmu.2017.01091

**Published:** 2017-09-04

**Authors:** Ayooluwa O. Douglas, David R. Martinez, Sallie R. Permar

**Affiliations:** ^1^Duke Human Vaccine Institute, Durham, NC, United States; ^2^Department of Molecular Genetics and Microbiology, Duke University Medical Center, Durham, NC, United States; ^3^Department of Pediatrics, Duke University Medical Center, Durham, NC, United States

**Keywords:** mother-to-child transmission, HIV, vaccines, vertical HIV transmission, neutralizing antibodies, non-neutralizing antibodies, maternal vaccines

## Abstract

Despite the wide availability of antiretroviral therapy (ART) prophylaxis during pregnancy, >150,000 infants become infected through mother-to-child transmission (MTCT) of HIV worldwide. It is likely that additional intervention strategies, such as a maternal HIV vaccine, will be required to eliminate pediatric HIV infections. A deeper understanding of the fine specificity and function of maternal HIV envelope (Env)-specific responses that provide partial protection against MTCT will be critical to inform the design of immunologic strategies to curb the pediatric HIV epidemic. Recent studies have underlined a role of maternal HIV Env-specific neutralizing and non-neutralizing responses in reducing risk of MTCT of HIV and in prolonging survival rates in HIV-infected infants. However, critical gaps in our knowledge include (A) the specific role of maternal autologous-virus IgG-neutralizing responses in driving the selection of infant transmitted founder (T/F) viruses and (B) Env mechanisms of escape from maternal autologous virus-neutralizing antibodies (NAbs). A more refined understanding of the fine specificities of maternal autologous virus NAbs and ways that maternal circulating viruses escape from these antibodies will be crucial to inform maternal vaccination strategies that can block MTCT to help achieve an HIV-free generation.

## Introduction

According to the 2016 UNAIDS global report, >150,000 infants became infected with HIV-1 *via* mother-to-child transmission (MTCT) in 2015 (1). This is despite the great success in expanding the availability of antiretroviral therapy (ART) worldwide. Ongoing challenges for the elimination of pediatric HIV infection include the following: lack of universal HIV testing and treatment during pregnancy, late maternal presentation for clinical care, maternal HIV acquisition in late pregnancy, and lack of maternal adherence to ART therapy during breastfeeding (1, 2). Thus, it is likely that alternative strategies, such as a maternal or infant HIV vaccine, will be required to eliminate pediatric HIV infections.

Mother-to-child transmission of HIV can occur *via* three distinct routes: during pregnancy (antepartum), during labor and delivery (peripartum), and during breastfeeding (postpartum). Maternal ART has been highly successful in reducing MTCT of HIV to as low as 2% transmission risk; however, poor maternal adherence to ART therapy, ART-associated toxicity in infants, and limited ART availability in resource-limited areas remain outstanding challenges in preventing MTCT of HIV (2). Interestingly, in the absence of maternal ART prophylaxis during pregnancy or at delivery, only 30–40% of HIV-infected mothers vertically transmit HIV to the infant, suggesting that maternal factors may provide partial protection against vertical transmission of HIV infection (2, 3). These factors could include maternal immune responses capable of mediating partial protection against MTCT of HIV. Efforts to develop immune-based strategies that can synergize with current ART prophylaxis to further reduce MTCT risk have focused on understanding the role of maternal HIV envelope (Env)-specific antibodies in mediating protection against HIV transmission. Interestingly, maternal IgG is transferred to fetus across the placenta throughout gestation and mediates protection against neonatal infections during the first few months of life (4–6). Given that maternal HIV Env-specific IgG responses are present at the time of infant infection, MTCT provides a unique setting to elucidate the role of maternal passively acquired Env-specific IgG in mediating protection against virus acquisition in infants. Understanding the determinants of how infant T/F viruses initiate infection in the presence of maternal neutralizing and non-neutralizing antibodies (NAbs) in the fetus could also shed light on mechanisms of virus superinfection in adults. Furthermore, defining viral escape mechanisms from autologous virus NAbs is not only relevant in the setting of MTCT but may also have important applications in our understanding of more general virus escape mechanisms from host immune responses.

## The Role of Maternal NAbs in MTCT

The role of maternal autologous virus NAbs in protecting the neonate against HIV transmission remains unclear. Although some studies reported higher levels of NAbs in serum among non-transmitting mothers (7–10), other studies have not confirmed this association (11–13). These contradictory results may be due to the small mother infant pair sample sizes, unclear timing of infant HIV-1 infection, disparate maternal and infant sample collection times, unknown route of vertical transmission, ART prophylaxis or treatment during pregnancy, delivery or postpartum, and lack of control for factors that impact MTCT, such as maternal plasma viral load and peripheral CD4+ T cell count (14, 15). Given the extensive genetic and antigenic diversity of HIV within a host, elucidating the fine specificity of maternal NAb responses against conserved vulnerable regions of the HIV Env may provide a better understanding of maternal immune correlates of protection against MTCT. The conserved vulnerable regions on the HIV Env include the following: the CD4 binding site, the variable loop regions 1 and 2 (V1V2), variable loop region 3 (V3), and the gp41 membrane-proximal external region (MPER) (16).

Our group recently identified maternal humoral correlates of protection against peripartum HIV transmission in a large cohort of *n* = 248 HIV-infected women from the Women and Infant Transmission Study (WITS), a historic North American observational cohort of HIV clade B virus-infected, ART naïve pregnant women (9). Importantly, this maternal humoral correlates of protection analysis accounted for known maternal MTCT risk factors such as maternal plasma viral load, peripheral CD4+ T cell count, infant gestational age, and delivery mode, thereby isolating the role of maternal HIV Env-specific IgG responses in mediating partial protection against MTCT of HIV. In the WITS cohort, maternal V3-specific IgG binding responses, tier 1 virus-neutralizing responses, and CD4 binding site-blocking responses all correlated and were independently predictive of reduced MTCT risk (9). Further analyses of the fine specificity and function of the potentially protective maternal V3-specific IgG responses revealed that both binding and neutralizing responses targeting the C-terminal region were associated with reduced MTCT risk (8). Thus, these findings provide proof of principle that maternal HIV Env-specific IgG responses targeting vulnerable epitopes on the HIV Env can afford partial protection against peripartum MTCT of clade B HIV. Furthermore, a separate study observed that Env-specific responses targeting MPER region in gp41 were associated with reduced MTCT risk (17), suggesting that multiple regions in HIV Env may be targets of potentially protective maternal humoral responses. However, the association of maternal humoral responses to defined vulnerable Env epitopes with reduced vertical virus transmission has not been consistently confirmed in other cohorts of HIV-infected women. For example, the association of maternal V3-specific IgG binding responses, tier 1 virus-neutralizing responses, and CD4 binding site-blocking responses were not predictive of reduced MTCT risk in HIV, clade C virus-infected women from the Breastfeeding and Nutrition (BAN) cohort (*n* = 88) (18). However, it should be noted that the majority of transmitting women included in the BAN humoral correlates of protection analysis transmitted *in utero* and in the setting of maternal ART treatment. Nevertheless, these findings suggest that maternal humoral correlates of protection against MTCT of HIV in ART naïve, clade B virus-infected women may not be applicable to other transmission modes (i.e., *in utero* transmission), other viral clades, and/or in the setting of maternal ART treatment. Further defining the fine specificity and function of potentially protective maternal humoral responses will provide immunologic benchmarks used to evaluate future maternal HIV vaccine modalities that may temporarily enhance virus blocking antibody responses during pregnancy. For example, in the moderately protective RV144 vaccine efficacy trial, vaccine-elicited V1V2-specific IgG responses were associated with reduced HIV transmission risk, and thus the elicitation of V1V2-specific IgG responses is currently being used as an immune benchmark in ongoing vaccine efficacy studies (19, 20). Furthermore, given that the fetus is passively immunized with maternal IgG throughout pregnancy, a deeper understanding of the role of NAbs that are present in a host at the time of infection could help inform vaccine strategies.

## Transmitted Founder (T/F) Viruses That Infect Infants and Their Sensitivity to Maternal Antibodies

**Figure 1:**
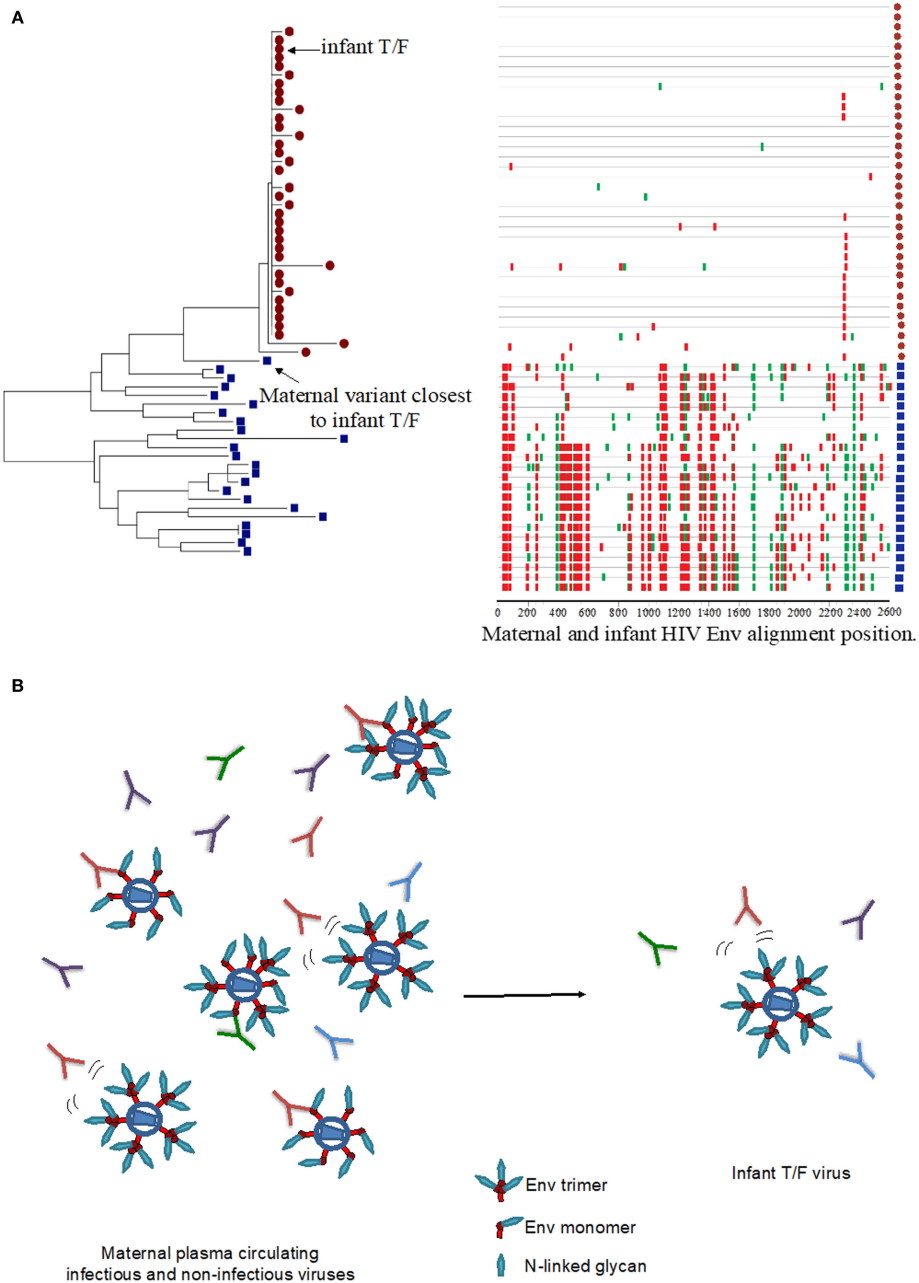
HIV virus escape from maternal neutralizing antibodies (NAbs) in the setting of mother-to-child transmission. **(A)** Neighbor joining phylogenetic tree and highlighter plot of the full HIV envelope (Env) gene (*env*) for one mother–infant pair, showing the transmission of one T/F virus from mother to infant. The red circles represent infant *env* amplicons, and the blue squares represent maternal *env* amplicons in the highlighter plot. Red ticks represent non-silent amino acid mutations, and green ticks represent silent amino acid mutations in the HIV Env region. Neighbor-joining tree was generated using MEGA7, and the highlighter plot was generated using the Los Alamos National Laboratory HIV tools: highlighter plot. **(B)** Maternal infectious and non-infectious virus quasispecies in the presence of a wide pool maternal autologous-virus NAbs may select for infectious neutralization-resistant viruses that infect the infant.

Similar to HIV infection in adults, HIV-infected infants become infected with one to a few HIV viruses, suggesting that a selective virus genetic bottleneck is involved in MTCT (Figure 1A) (7, 11, 12, 14, 15). However, factors that drive this selective virus genetic bottleneck are not clear. Env-specific IgG responses can mediate immune pressure on autologous circulating viruses and therefore could contribute to the selection of infant T/F viruses (Figure 1B). While some studies have suggested that viruses transmitted from mother to infant may be resistant to neutralization by maternal antibodies (10, 21, 22), other studies have not confirmed these observations (13, 23). The reported increased resistance of infant T/F viruses to maternal NAbs may be explained by genetic differences compared to maternal non-transmitted viruses at key sites including Env glycan motifs. Furthermore, mutation of distal amino acid residues relative to Env neutralizing epitopes could also confer neutralization resistance to maternal autologous virus NAbs (24) (Figure 1B). A recent study in HIV, clade A virus-infected women examined the neutralization sensitivity of maternal autologous circulating viruses to paired plasma in 10 transmitting and 10 non-transmitting women and found no association in autologous virus-neutralizing activity and transmission risk (23). This study also reported that transmitting and non-transmitting women had a similar proportion of neutralization-resistant viruses to paired maternal plasma, suggesting that maternal autologous NAbs may not be associated with infant protection. However, to date, no study has evaluated whether neutralization resistance to paired maternal plasma NAbs is a defining feature of infant T/F viruses compared to maternal non-transmitted variants. Given that maternal autologous virus NAbs will only need to block the viruses that initiate infection in the infant (i.e., infant T/F viruses), future studies should focus on defining the susceptibility of infant T/F viruses to paired maternal plasma NAbs and monoclonal NAbs with defined epitope specificities compared to non-transmitted maternal variants in a cohort with standardized sample collection and known transmission risk factors.

In contrast to adult HIV transmission in which an HIV vaccine will need to elicit broadly NAbs against difficult-to-neutralize viruses from several clades, MTCT is a unique setting in which vaccine-elicited antibody responses need to only block the maternal virus pool to which the infant is exposed to (Figure 1B). Therefore, vaccination strategies aimed at eliciting broadly NAbs against multiple viral clades may be distinct from immunization strategies aimed at the inducing autologous virus NAbs against a defined and limited pool of maternal viruses. As a maternal and/or infant HIV vaccine will most likely be necessary for eliminating pediatric HIV infections, identifying the maternal NAbs that target specific vulnerable Env epitopes in selecting for neutralization-resistant viruses will be important to inform maternal vaccination strategies. Moody et al. recently demonstrated that in an HIV-infected individual, autologous-virus NAbs targeting the V3 loop and CD4 binding site neutralized a large proportion of autologous viruses isolated from plasma (25). Importantly, the autologous virus NAbs in this individual mediated the neutralization of heterologous easy-to-neutralize tier 1 virus isolates but failed to neutralize difficult-to-neutralize heterologous tier 2 virus isolates, suggesting that these seemingly inconsequential weakly NAbs can drive the selection of predominant strains that repopulate the autologous virus pool in HIV-infected individuals. This observation underlines the role of maternal Env-specific NAbs in selecting for neutralization-resistant viruses circulating in the blood. In the setting of MTCT, these maternal plasma tier 1 virus NAbs could select for neutralizing resistant viruses in the maternal blood compartment, and these viruses may be transmitted to the infant. Thus, maternal V3 and CD4bs-specific NAbs may select for maternal autologous circulating viruses that are neutralization resistant and may drive the selection of infant T/F viruses. Therefore, it will be important to define both the fine specificity and neutralizing function of maternal autologous virus NAbs.

## The Role of Maternal Non-Neutralizing Humoral Responses and MTCT Risk

The role of maternal non-neutralizing humoral responses in mediating partial protection in the setting of MTCT of HIV also remains unclear. A study reported that in a small cohort of (*n* = 19) HIV clade A virus-infected Kenyan women, breast milk Env-specific IgG responses with antibody-dependent cellular cytotoxicity (ADCC) activity were associated with reduced MTCT risk (26). Interestingly, these ADCC-mediating IgG responses in breast milk were found to have limited neutralizing activity, suggesting that maternal ADCC responses may be important in limiting postpartum transmission of HIV. However, it should be noted that these findings were from a small cohort of 9 transmitting and 10 non-transmitting HIV-infected women and have not been validated in a larger cohort of clade A HIV-infected women. Pollara et al. examined the role of maternal Env-specific IgG responses in breast milk and found no association of ADCC-mediating responses and decreased MTCT risk in a cohort of (*n* = 87) of HIV clade C infected breastfeeding Malawian women (27). The inability to validate the association of maternal ADCC-mediating breast milk Env-specific IgG responses and reduced MTCT risk may be due to distinct cohort sizes, potential virologic differences in clade A and clade C viruses, and differences in fine specificity and function in these distinct cohorts of HIV-infected women. Despite the seemingly contradictory findings of the role of breast milk Env-specific ADCC-mediating IgG responses and postpartum MTCT risk, maternal passively acquired ADCC-mediating IgG responses have been associated with reduced infant mortality in HIV clade A, peripartum-infected infants, suggesting that maternal passively acquired ADCC responses may prolong infant survival in pediatric HIV-infected patients (28). Together, these studies highlight the potentially protective role of maternal ADCC-mediating Env-specific IgG responses and their importance in increasing infant survival rates in HIV-infected pediatric patients.

## The Transplacental Transfer of Maternal HIV Env-Specific IgG Responses and MTCT Risk

In the setting of pregnancy, maternal IgG is passively transferred to the fetus throughout gestation, with the majority of the transplacental transfer taking place in the third trimester (29). However, in the setting of maternal HIV infection, the transplacental transfer of maternal IgG to the fetus is poorly efficient (29–34). Despite the observed poor transplacental transfer of maternal IgG responses to the fetus in the setting of maternal HIV infection, maternal Env-specific IgG neutralizing responses may be efficiently transferred to the infant (35). However, the efficient transplacental transfer of maternal HIV Env-specific IgG neutralizing responses has not been found to be associated with decreased MTCT risk (35). As the role of maternal HIV Env-specific IgG in mediating infant protection against HIV infection remains unclear, it is not known if the poor transplacental transfer of potentially protective maternal Env-specific IgG responses leads to increased infant HIV transmission risk. However, some studies suggest that the transplacental transfer of maternal Env-specific IgG responses with antiviral functions may be important for infant protection (17). Passively acquired maternal IgG responses in HIV-exposed uninfected infants have been shown to mediate virus transcytosis inhibition *in vitro* in clade C HIV-infected mothers and their infants (17). Furthermore, the fine specificity of passively acquired maternal Env-specific IgG responses was mapped to gp41 epitopes that encompass the MPER, a key site that is commonly targeted by broadly NAbs. Thus, the transplacental transfer of maternal HIV Env-specific IgG responses with antiviral functions may be important for infant protection, as well as survival outcome upon infection (17, 28).

## Conclusion

It is likely that additional immune-based strategies such as a safe and effective maternal and/or infant HIV-1 vaccine that can synergize with current prophylactic ART treatments will be required to eliminate pediatric HIV infections. Given the growing body of evidence on the role of maternal HIV Env-specific IgG responses and their association with reduced MTCT risk, more studies are needed to further refine the molecular details by which HIV viruses escape maternal NAbs. Larger and better controlled studies that investigate maternal NAbs with defined fine-epitope specificity and their role in preventing or reducing MTCT risk in the setting of ART may provide crucial information for the design of an effective maternal and/or infant HIV-1 vaccine to help achieve an HIV-free generation.

## Author Contributions

DM and SP conceived the topic. AD, DM, and SP wrote the manuscript and AD prepared the figures.

## Conflict of Interest Statement

The authors declare that the research was conducted in the absence of any commercial or financial relationships that could be construed as a potential conflict of interest.
